# Rare tumors of the heart–angiosarcoma, pericardial lipoma, leiomyosarcoma, three case reports

**Published:** 2010-05-25

**Authors:** I Stoian, I Tepes Piser, I Kulcsar, O Chioncel, A Carp, C Macarie

**Affiliations:** ‘Prof Dr CC Iliescu’ Institute of Cardiovascular Diseases, BucharestRomania

**Keywords:** cardiac tumors, cardiac angiosarcoma, pericardial lipoma, leiomyosarcoma

## Abstract

Primary tumors of the heart, pericardium and inferior vena cava are extremely rare. Three cases of surgically / biopsy proven angiosarcoma of the right atrium, pericardial lipoma and leiomyosarcoma of inferior vena cava–demonstrated by ultrasound, computed tomography (CT) and magnetic resonance imaging (MRI)–are presented here.

## Case 1. Cardiac angiosarcoma

Primary cardiac sarcoma is a rare clinical entity (20% of all cardiac neoplasms) and 0.0001% in collected autopsy series [1,2]. The biological behavior of cardiac sarcomas is similar to all soft–tissue sarcomas (mesenchymal tissue) [1]. Angiosarcomas are the most common cardiac sarcomas and make up 33% of the cases [2].

The medial survival of these aggressive tumors is of only 6 months [1,2]. The clinical picture is nonspecific and appears late in the evolution of the disease. Signs of right heart failure are frequently being described. Standard surgery, adjuvant chemotherapy and radiotherapy have been unsuccessful.

We present the case of a 45–year–old woman with severe progressive exertional dyspnea. She was found to have a large cardiac mass in the right atrial cavity, and through the tricuspid valve, extanding into the right ventricular inflow, on the transthoracic echocardiography. The tumor seems to have a large origin in the posterior and lateral free right atrial wall. The patient underwent sternotomy, the mass was found in the lateral and posterior right atrial wall, extanding upon the atrioventricular junction and into the anterior superior mediastinum. The biopsy revealed a high–grade angiosarcoma. Because of the severe bleeding, the excision was limited to a very small part of the tumor laying on the right atrial surface.

The patient succombed two days after the surgery because of severe pulmonary embolism. The autopsy and histologic examination of the tumor was positive for cardiac angiosarcoma.

**Figure 1:**
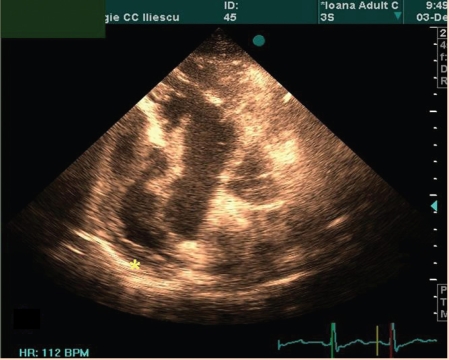
Apical four–chamber recording of a large right atrium and enlarged right ventricular cavity. Diastole before the atrial depolarization. The long masses arise in the posterior and lateral right atrial wall. (asterix)  The tumor extends into the atrial cavity and through the opened tricuspid valve, into the right ventricular inflow.

**Figure 2:**
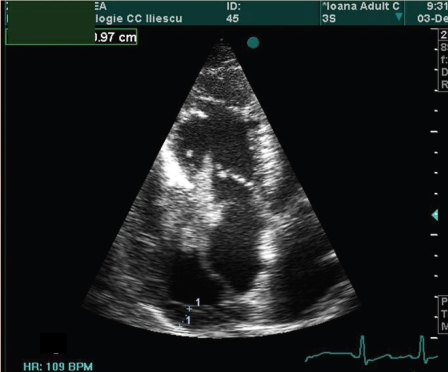
The same apical four–chamber plan. Ventricular systole. The tumor is inside the right ventricular cavity passing through the semiclosed tricuspid cusps.

**Figure 3:**
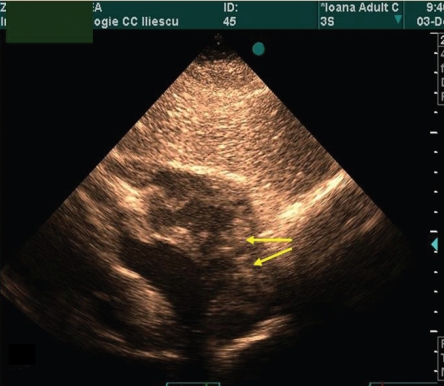
Subcostal apical four–chamber view. The right atrium is the anterior cavity. The arrows point at the multiple sites of tumor insertions in the right atrial wall.

**Figure 4:**
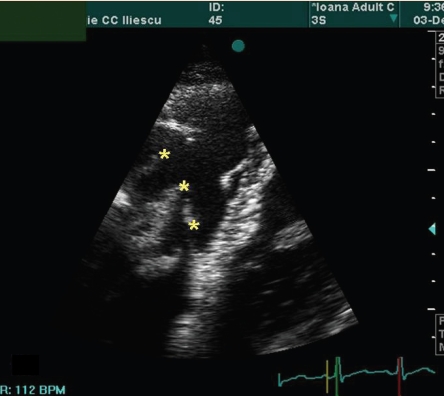
Apical four–chamber view. Diastole. Zoom of the masses (asterix) inside the right ventricular inflow, just under the tricuspid annular plane.

The echocardiography of the patient is presented in (Figure 1, Figure 2,Figure 3,Figure 4)

## Conclusion

Cardiac angiosarcomas are rare entities, very aggressive and with highly metastatic spreading. According to the cardiac site, there are right heart angiosarcomas (presented case), left heart angiosarcomas and pulmonary artery angiosarcomas [1,2]. The histologic type affects the prognosis and the survival rate. The tumor excision, associated with chemotherapy and radiotherapy do not enhance the life expectancy.

## Case 2. Leiomyosarcoma of the inferior vena cava extanding into the right cardiac chambers

Leiomyosarcomas (malignant retroperitoneal) are the second most common retroperitoneal neoplasms in adults. Leiomyosarcomas of the inferior vena cava are rare clinical entities, described in less than 300 patients [5]. This rare neoplasm involves the uterus, gastrointestinal tract and the skin; retroperitoneal localization is rare, in particular inferior vena cava involvement.

We present the case of a 42–year–old woman, investigated for pain in the upper right abdominal quadrant; no other symptoms were described. Abdominal ultrasound demonstrated the presence of an echogenic mass, of 5 x 7cm, in the retroperitoneal space, behind the right kidney. The mass appears to originate from the inferior vena cava. The echocardiographic exam of the right atrium cavity and the oriffice of the inferior vena cava (subcostal area) revealed a long mass entering from vena cava into the right atrium cavity, and through the tricuspid orifice, in the right ventricle cavity. The echostructure was thin, homogenous, and the long mass slightly moved along with the heart. CT and MRI confirmed the presence of the retroperitoneal tumor and the origin in the inferior vena cava.

Surgery was performed and a mass of 6x 8x 3 cm was found, spreading out from middle vena cava. Part of the tumor was excised, including that from the vena cava. At biopsy –leiomyosarcome. Adjuvant radiation therapy was planned. The evolution remained unknown because of the patient's lack of presentation.

Due to difficult images recording, we only present one echocardiographic shot, recorded at the admission.

**Figure 5:**
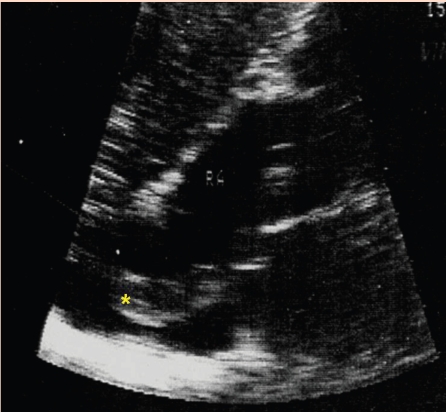
Subcostal recording of the right atrium cavity and  the vena cava entering the atrial cavity. A long mass (asterix) inside the vena cava and right atrial cavity, passing into the tricuspid oriffice and the right ventricular inflow.

## Conclusion

The leiomyosarcomas are rare entities. The tumors are slowly–growing, well encapsulated, tending to grow by expansion [5]. The tumors are classified in relation to the renal and hepatic veins. So, 34% occured in Segment Ⅰ (from the renal to hepatic veins), 41.7% in Segment Ⅱ (infrarenal) and 24.3% in Segment Ⅲ (from hepatic vein to the vena cava and right atrium) [6]. Surgical removal is the treatment of choice.

## Case 3. Pericardial lipoma

Pericardial lipomas are rare clinical findings – account for 10% of all primary cardiac tumors  [4]. We describe a case of pericardial /epicardial lipoma.

A 45–year–old female was admitted due to cardiomegaly of unknown etiology. The 2D echocardiography showed a large epicardial mass located along the anterolateral surface of the heart, from the midlateral left ventricular wall level to the midlateral left atrium wall level. The mass showed an echostructure identical to that of subcutaneous adipose tissue. The  thoracic CT exam confirmed the presence of the pericardial tumor, adipose like structure.

The histologic examination of the tissue samples obtained by biopsy while the surgery, revealed mature adipose tissue. The adipose mass was encapsulated, weighing 386g; the tumor origin was at the left ventricular lateral wall epicardium and visceral pericardium with invasion of the ventricular wall. The tumor was completely excised. The microscopic examination confirms the diagnosis of lipoma. The patient had an uneventful postoperative recovery. In the late postoperative period (three months after the surgery procedure) the patient had one presyncope, ventricular tachycardia, on electrocardiography (standard, Holter 24h). Amiodarone stopped the ventricular arrhythmia. After 24 months, the patient was asymptomatic and had no evidence of reccurence (on clinical and echocardiographic exams).

**Figure 6:**
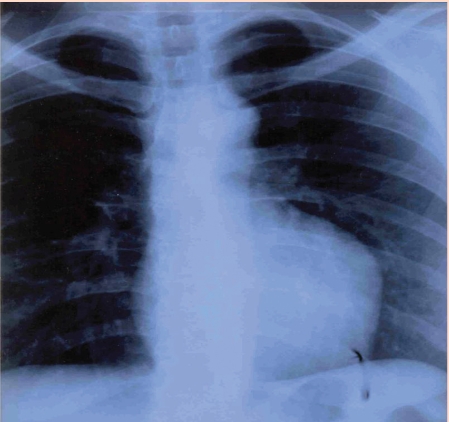
PA thoracic X– ray. Abnormal cardiothoracic index. Abnormal cardiac silhouette. Enlarged left cardiac border

**Figure 7:**
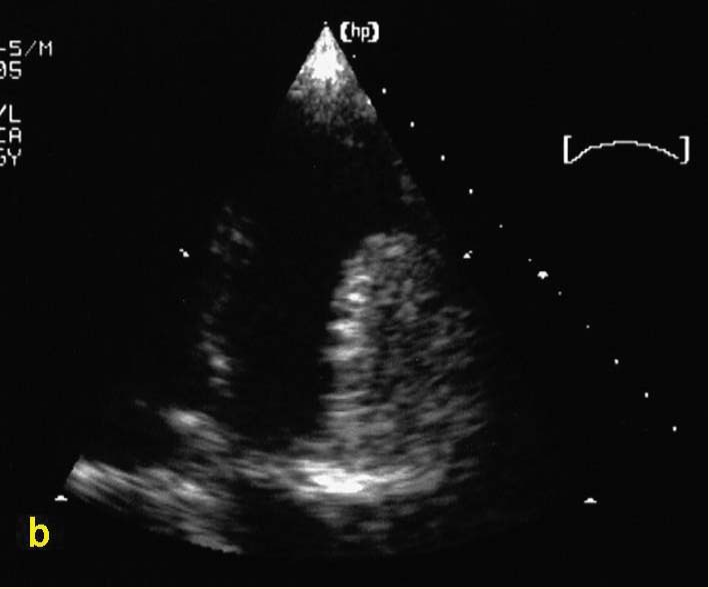
Apical four–chamber view. The ovoid tumor – homogenous and intense echostructure, near the mid / base lateral left ventricular wall and the proximal lateral left atrial wall. The tumor seems to bulge into the left ventricular cavity

**Figure 8:**
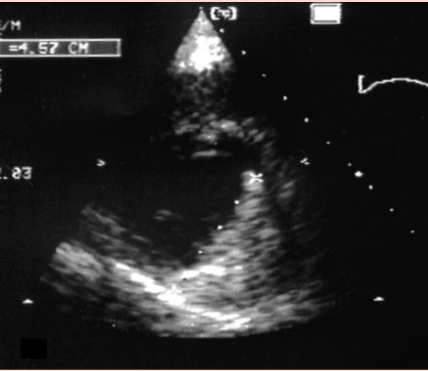
Parasternal short axis recording of the left ventricular cavity. The tumor– semilunar shape, extended from one o'clock to seven o'clock. The same homogenous, uniform echostructure of the mass

**Figure 9:**
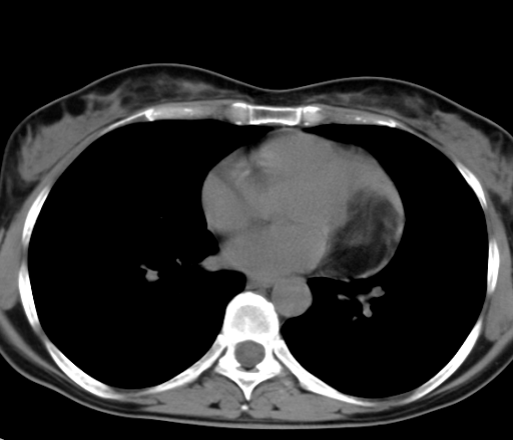
Thoracic CT scan. The large, ovoid tumor, 3cm/5cm in axial plan and 4 cm in cranio–caudal plan. Located in the intrapericardial space, along the left border of the heart, towards the base of the heart.

**Figure 10:**
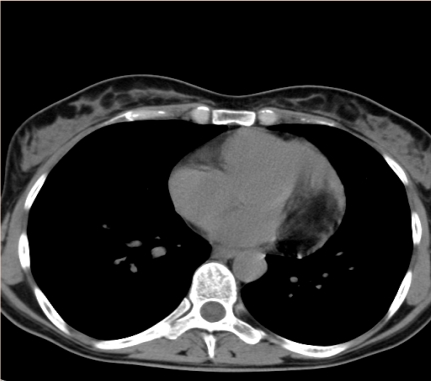
Thoracic CT scan. The large, ovoid tumor, 3cm/5cm in axial plan and 4 cm in cranio–caudal plan. Located in the intrapericardial space, along the left border of the heart, towards the base of the heart.

**Figure 11:**
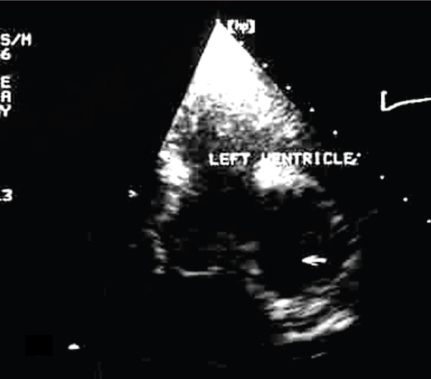
Apical four–chamber recording of the left ventricle and left atrium after surgery. An ovoid echo–free space (arrow)– the pericardial cavity after the tumor was removed.

**Figure 12:**
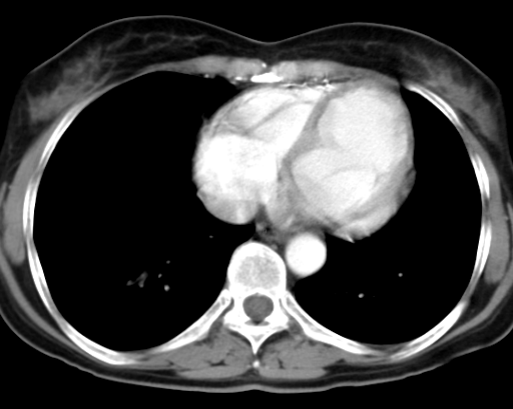
Thoracic CT scan after the tumor was excized. Normal heart. No pericardial free space in this section plan.

**Figure 13:**
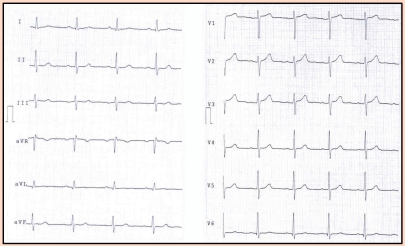
Standard 12 leads electrocardiography recorded before surgery. Normal sinus rhythm; 62 bpm.

**Figure 14:**
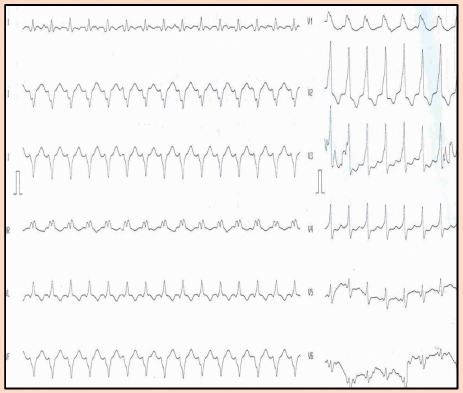
Standard 12 leads electrocardiography after the tumor excision. Presyncope before this recording. Paroxistic tachycardia, 180 bpm with wide QRS  and RBBB morphology. Left ventricular origin may be the site of the tachycardia.

**Figure 15:**
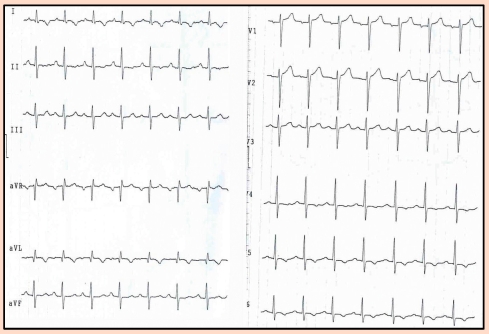
Electrocardiography recorded 10 min after the cessation of the tachycardia. Sinus rhythm, 100 bpm. Inversed T wave in the left precordial leads.

## Conclusion

Pericardial lipomas are rare. The tumors are best diagnosed on imaging tests including echocardiography, CT, MRI. The only therapeutic method is complete surgical excision.
